# Higher general executive functions predicts lower body mass index by mitigating avoidance behaviors

**DOI:** 10.3389/fendo.2022.1048363

**Published:** 2022-11-09

**Authors:** Marco La Marra, Ciro Rosario Ilardi, Ines Villano, Rita Polito, Maria Raffella Sibillo, Marina Franchetti, Angela Caggiano, Francesca Strangio, Giovanni Messina, Vincenzo Monda, Girolamo Di Maio, Antonietta Messina

**Affiliations:** ^1^ Department of Experimental Medicine, University of Campania “Luigi Vanvitelli”, Naples, Italy; ^2^ Department of Psychology, University of Campania “Luigi Vanvitelli”, Caserta, Italy; ^3^ Department of Clinical and Experimental Medicine, University of Foggia, Foggia, Italy; ^4^ Department of Movement Sciences and Wellbeing, University of Naples “Parthenope”, Naples, Italy

**Keywords:** obesity, executive functions, BMI – body mass index, body image, avoidance

## Abstract

**Background:**

The present study examines the relationship between obesity, executive functions, and body image in a nonclinical population from southern Italy.

**Methods:**

General executive functioning (Frontal Assessment Battery–15), and body image disturbances (Body Uneasiness Test) were assessed in a sample including 255 participants (138 females, M age = 43.51 years, SD = 17.94, range = 18–86 years; M body mass index (BMI) = 26.21, SD = 4.32, range = 18.03–38.79).

**Findings:**

Multiple Linear Regression Analysis indicated that age, years of education, FAB15 score, body image concerns, and avoidance predicted the variance of BMI. A subsequent mediation analysis highlighted that the indirect effect of FAB15 on BMI through avoidance was statistically significant.

**Interpretation:**

Our results suggest that more performing executive functioning predicts a decrease in BMI that is partially due to the mitigation of avoidance behaviors.

## Introduction

Obesity is a chronic disease and a major public health challenge (1). In recent decades, it has been argued that excess body fat, in addition to being an important risk factor for many chronic diseases (2, 3), could represent a significant predictor of impaired cognitive performance, accelerated cognitive decline, and dementia (4–8). In this vein, excess body weight appears to be related to reduced brain volume in cognitively healthy older individuals, but in patients with cognitive impairment it may exert an additional detrimental effect (9). More specifically, increased body adiposity has been found to correlate with generalized structural alterations involving the orbital frontal cortex, temporal and parietal cortices, and hippocampus (10–12). The first generation of studies investigating the relationship between obesity and cognition found that obese subjects performed worse on tasks assessing global cognitive functioning, particularly impacting attention and memory (13, 14). However, more recently, attention has focused on more targeted cognitive abilities, such as executive functions (EFs) (15–20). Functionally related to the integrity of the prefrontal cortex, EFs are an umbrella term encompassing different higher order cognitive domains (e.g., abstraction, cognitive flexibility, inhibitory control, working memory, and planning) that are crucial for the guidance, direction and management of the general cognition, emotions, and goal-directed behaviors (21, 22). They enable individuals to respond adaptively to environmental demands, especially in conflict and unfamiliar contexts (23, 24). Therefore, it is expected that EFs play a critical role in modulating eating behavior.

It has been argued that EFs could predict body weight variability (18, 25). For instance, EFs were found to be related with increased intake of high-fat foods (26–31), poor energy expenditure (32), increased susceptibility to emotional eating (33), inability to learn from past experiences (34), and worse outcomes in treatments aimed at weight decrease (27, 35). In addition, neuroimaging evidence has highlighted a decreased neural activity in the prefrontal cortex of obese subjects (36–38). Nevertheless, conflicting results are also available. In fact, some studies have shown that executive performance of obese subjects were equal or better than those of normal-weight subjects (39–46). These observations support the “obesity paradox” (47–50), which lies in the hypothesis that obesity could play a protective role for health status, especially in the elderly population. Since several covariates could affect the relationship beween EFs and obesity (46, 51), this association deserves to be further investigated (52).

An interesting line of research has focused on the link between eating habits and body image disturbances in obesity (53, 54). Similar to patients with eating disorders, patients with obesity exhibit excessive body dissatisfaction (55), undue weight-related concerns (56–59), and body image distortion (60–62). Body image refers to a multidimensional construct underlying how individuals perceive, think, and feel about their bodies (63). It is usually assessed along a continuum ranging from unhealthy body perceptions (inaccurate perceptions and major negative qualities) to healthy body perceptions (accurate perceptions and predominantly positive attributes) (63, 64). It has been shown that body image-related dissatisfaction may predict the onset of psychopathological symptoms (including depression and low self-esteem) and unhealthy behaviors (e.g., smoking, unhealthy weight control behavior) (65, 66). According to cognitive theories, schemas related to appearance, body shape and body weight impact on body image (67, 68). Negative body image perception would be associated with biases that affect selective attention, information processing, memory, and reasoning/judgment skills. Such biases allegedly increase negative emotions resulting from body image dissatisfaction, which would promote unhealthy behaviors aimed at changing body shape and weight, such as eating disorders (68, 69). Analysis of cognitive biases has shown that attentional biases would underlie psychopathology; however, memory and judgment biases –the degree to which emotionally salient stimuli are encoded, recalled, perceived, and processed– could also play an important role in behavioral disorders (68, 69). Results from eye-tracking studies have suggested that individuals with high levels of body dissatisfaction tend to orient their attention more towards stimuli related to desired (70, 71) and feared (72, 73) appearance. Individuals with higher levels of body dissatisfaction seem to show greater attentional bias towards negative appearance-related (74, 75) as well as toward positive appearance-related stimuli (75–78). Some studies on patients with eating disorders have highlighted that subtle cognitive dysfunctions could alter body schema information processing. These might depend on the interaction between bottom-up and top-down mechanisms (79–81), in which frontal circuits that support EFs are involved. This interaction appears to be supported by neuroimaging evidence that detected the involvement of the limbic/paralimbic system, medial prefrontal cortex (mPFC) (82–84) and parietal lobe (85, 86) in patients with eating and weight disorders (EWDs) during self-image exposure tasks.

To date, a clear understanding of how EFs interact with body image perception in obese individuals is lacking. Thus, the aim of the present study was to offset this gap within the scientific literature, exploring the hypothesized relationship between EFs, body image and body weight in a nonclinical headings.

## Methods

### Participants

A convenience sampling method was used and data from 287 participants (155 females) were collected. Participants were Italian volunteers recruited across different districts of Southern Italy (i.e., Campania, Calabria, and Puglia regions). Inclusion criteria were as follows: age ≥ 18 years, formal schooling ≥ 5 years (i.e., primary school), and adjusted score greater than the normative cutoff (i.e., 23.80) on the Mini-Mental State Examination (MMSE) (87, 88). Exclusion criteria were as follows: neurocognitive, psychiatric or psychopathological diseases, past or present intellectual and/or linguistic deficits, and presence of serious health conditions (e.g., cancer or morbid obesity). None of the participants had history of alcohol or substance abuse/addiction and was treated with drugs interfering with cognitive functioning. Individuals with well-compensated chronic medical illnesses such as hypertension, type II diabetes, or cardiovascular diseases were not excluded to prevent the construction of a hyper-normal sample.

### Materials and procedure

Participants were tested individually in a sound-proofed room. After acquisition of sociodemographic data (i.e., sex, age, and education), body mass index (BMI) was calculated according to Quetelet’s formula (kg/m^2^). Following the administration of MMSE, participants were administered the Frontal Assessment Battery-15 (FAB15) (89, 90) and the Body Uneasiness Test-Form A (BUT-A) (91). The FAB15 is a short neuropsychological screening battery employed to assess general executive functioning. It consists of five subtests exploring abstraction abilities, generativity, planning, sensitivity to interference, and inhibitory control. The scoring range is 0–15, with a higher score reflecting a better performance. In a recent normative study on a large sample of healthy individuals, the FAB15 demonstrated good internal consistency (Cronbach’s alpha = 0.72), a solid factorial structure (explained variance ranging from 53.80 to 73.79), and excellent interrater and test–retest reliabilities (90). The BUT-A is a 34-item self-report measures used to perform a multidimensional clinical assessment of body uneasiness. It simultaneously explores different areas of body-related psychopathology: dissatisfaction about the body and its weight; avoiding and compulsive control behavior; experience of separation and foreignness regarding the body; specific worries for certain body parts, characteristics or functions. It consists of five subscales: weight Phobia (WP, 8 items), body image concerns (BIC, 9 items), avoidance (AV, 6 items), compulsive self-monitoring (CSM, 5 items), and depersonalization (D, 6 items). The global severity index (GSI) is the average rating of all the 34 items. The BUT-A shows good internal consistency (Cronbach’s = 0.79–0.90), good test-retest reliability (correlation coefficients greater than 0.7), and satisfactory concurrent and discriminant validity (91). Participants were not aware of the specific aims of the study in order not to influence their response to the self-administered tests.

### Ethics statement

All participants gave prior written informed consent to the study which was approved by the ethics committee of the University of Campania “Luigi Vanvitelli” and carried out according to the 1964 Declaration of Helsinki.

### Statistical analyses

Statistical analyses were conducted in line with the generalized linear model. Descriptive statistics were expressed as frequency for nominal variables and mean and standard deviation for continuous variables. A multiple linear regression analysis (simultaneous entering method) was performed loading BMI as dependent variable and sex, age, education, FAB15 score, and BUT-A scores as predictors. The variance inflation factors (VIFs) and tolerance values were computed for determining the presence of a statistically significant multicollinearity (VIF = 1, no collinearity; VIF = 1 to 5, moderate collinearity; VIF > 5, high collinearity). Tolerance values ≥ 0.10 were deemed acceptable (92, 93). To establish whether, and how much, body image subdomains mediated the relationship between executive functioning (i.e., FAB15) and BMI, a mediation analysis was performed in line with results from multiple regression. To evaluate the significance of direct and indirect effects, bootstrapping procedure with 5,000 samples with replacement from the full sample to construct bias-corrected 95% confidence intervals (95% CI) was used (94). Statistical analyses were performed by means of IBM SPSS Statistics v. 26. Particularly, the SPSS Macro PROCESS was applied to execute mediation analysis. Finally, G*Power 3.1.9.4 was used to perform power analysis. A p-value ≤ 0.05 was considered statistically significant.

## Results

### Preliminary data analysis: normality assumptions and missing data

Univariate normality was assessed according to skewness and kurtosis indexes. More specifically, values ranging from -2 and +2 indicate the absence of appreciable deviations from normality. Square root transformation (√X_i_) was applied to normalize variables in line with skewness parameter (|1|< γ< |2|). Univariate outliers, i.e., z-scores higher than 3 in absolute terms (95), were deleted from the dataset (*n* = 32). For diagnostics of multivariate outliers, Mahalanobis’ distance $\left({\text{D}}_{i}^{2}\right)$ was calculated. No multivariate outliers were detected (mean ${\text{D}}_{i}^{2}$ = 11.91, df = 10, p_s_ > 0.001). Multivariate normality was assumed by Mardia’s coefficient $\frac{(\sum_{\text{i}=1}^{\text{N}}{\left({\text{D}}_{i}^{2}\right)}^{2}\;}{\text{N}})$ = 1.02< 120. Analysis of missing data showed a random missingness pattern (MCAR) that was handled *via* multiple imputation method.

### Descriptive statistics

Data from 255 volunteers (138 females) were analyzed (**Table 1**). Mean age of participants was 43.51 years (SD = 17.94, range = 18–86 years), whereas their mean education was 14.69 years (SD = 3.97, range = 5–20 years). Mean BMI value was 26.21 (SD = 4.32, range = 18.03–38.79; normal-weight, n = 101, mean BMI = 22.17, SD = 1.89; overweight, n = 98, mean BMI = 26.74, SD = 1.34; obese, n = 56, mean BMI = 32.56, SD = 2.33). On average, participants got a raw FAB15 score equal to 13.35 (SD = 1.55, range = 9–15). As for the BUT-A scores, mean values are the following: WP, mean = 1.32 (SD = 0.95, range = 0–3.88); BIC, mean = 1.06 (SD = 0.75, range = 0–3.33); AV, mean = 0.40 (SD = 0.50, range = 0–2.17); CSM, mean = 0.79 (SD = 0.54, range = 0–2.40); D, mean = 0.50 (SD = 0.44, range = 0–2.15). Mean GSI score was 0.87 (SD = 0.55, range = 0.03–2.59).

**Table 1:** Sample characteristics.

Variables	Descriptive statistics (N = 255)
Sex (females)	138
Age (years)	43.51 (17.94)
Education (years)	14.69 (3.97)
BMI (kg/m^2^)	26.21 (4.32)
FAB15 (raw score)	13.35 (1.55)
BUT-WP	1.32 (0.95)
BUT-BIC	1.06 (0.75)
BUT-AV	0.40 (0.50)
BUT-CSM	0.79 (0.54)
BUT-D	0.50 (0.44)
GSI	0.87 (0.55)

BMI, body mass index; FAB15, Frontal Assessment Battery-15; BUT-WP, Body Uneasiness Test-Weight Phobia; BUT-BIC, BUT-Body Image Concerns; BUT-AV, BUT-Avoidance; BUT-CSM, BUT-Compulsive Self-Monitoring; BUT-D, BUT-Depersonalization; GSI, Global Severity Index.

Mean (SD).

### Multiple linear regression analysis

Assumptions of linear regression analysis were satisfied. Particularly, the relationship between predictors and dependent variable was linear, no violations of homoscedasticity was detected, and the standardized residuals followed the normal distribution. Furthermore, results from power analysis (nominal α = 0.05, power = 0.80, effect size *f* ^2^ = 0.15, and number of predictors = 10) showed that a sample of 255 was more than acceptable (required sample size = 118). Due to abnormal VIF and tolerance values for the GSI score (VIF = 18.45, tolerance = 0.05), this variable was not included in the final model (see **Table 2**). The latter explained a significant amount of BMI variance (R^2^ = 0.46, F_(9, 246)_ = 20.057, p*<* 0.001). The variables found to be significant predictors of BMI were age (B = 0.05, p< 0.001), education (B = -0.44, p< 0.001), FAB15 (B = -0.37, p = 0.04), and both BIC (B = 3.30, p< 0.001) and AV subscales (B = 1.52, p = 0.04) of the BUT-A.

**Table 2:** Results of the Multiple Linear Regression Analysis on BMI.

Predictors	*B*	95% CI for *B*		*SE*	*t*	*p-*value
		LL	UL			
Sex	0.005	-0.769	0.779	0.393	0.013	0.990
Age	0.053	0.024	0.081	0.014	3.668	**<0.001**
Education	-0.437	-0.583	-0.292	0.074	-5.931	**<0.001**
FAB15	-0.367	-0.734	-0.002	0.186	-1.972	**0.039**
BUT-WP	-0.580	-1.350	0.190	0.391	-1.484	0.139
BUT-BIC	3.305	2.191	4.419	0.565	5.848	**<0.001**
BUT-AV	1.520	0.002	3.042	0.772	1.968	**0.041**
BUT-CSM	-0.954	-2.033	0.125	0.547	-1.743	0.083
BUT-D	0.737	-1.166	0.806	0.726	1.393	0.180

BMI, body mass index; FAB15, Frontal Assessment Battery-15; BUT-WP, Body Uneasiness Test-Weight Phobia; BUT-BIC, BUT-Body Image Concerns; BUT-AV, BUT-Avoidance; BUT-CSM, BUT-Compulsive Self-Monitoring; BUT-D, BUT-Depersonalization.

### Mediation analysis

**Figure 1:**
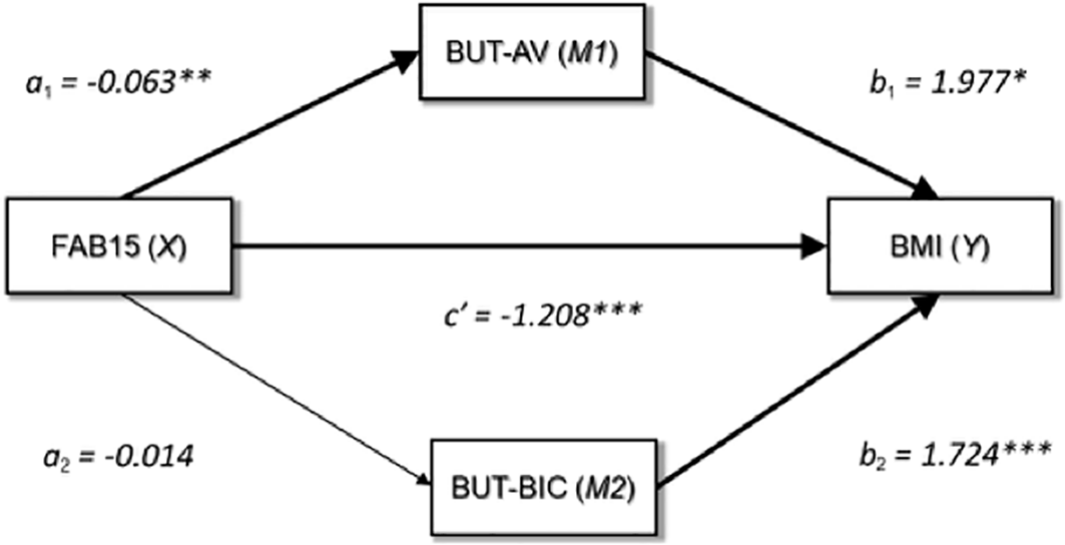
Graphical representation of the medication analysis output. FAB15, frontal assessment battery-15; BUT-AV, BUT-Avoidance; BUT-BIC, BUT-Body Image Concerns; BMI, body mass index; *p = <0.05; **p = <0.01; ***p = <0.001.

To test the mediating role of BUT-AV and/or BUT-BIC in the relationship between FAB15 (general executive functioning) and BMI, a mediation analysis was performed. A graphical representation of the mediation model is displayed in **Figure 1**. The FAB15 score was a significant predictor of BUT-AV (*a*_1_ = -0.063, 95% CI [-0.105, -0.022], SE = 0.021, *t* = -3.034, *p* = 0.002) but not of BUT-BIC (*a*_2_ = -0.014, 95% CI [-0.080, 0.051], SE = 0.033, *t* = -0.432, *p* = 0.666). However, both BUT-AV (*b*_1_ = 1.977, 95% CI [0.466, 3.489], SE = 0.767, *t* = 2.578, *p* = 0.010) and BUT-BIC (*b*_2_ = 1.724, 95% CI [0.780, 2.669], SE = 0.479, *t* = 3.599, *p*< 0.001) showed a positive association with BMI. As a result, the indirect effect of FAB15 on BMI *via* BUT-AV (*ab*_1_) was statistically significant (*ab*_1_ = -0.125, 95% CI [-0.305, -0.009], SE = 0.076) while the indirect effect through BUT-BIC (*ab*_2_) was not (*ab*_2_ = -0.025, 95% CI [-0.166, 0.091], SE = 0.123). The direct effect of FAB15 on BMI (*c′*) was significant (*c′* = -1.208, 95% CI [-1.550, -0.866], SE = 0.173, *t* = -6.965, *p*< 0.001); therefore, BUT-AV partially mediated the FAB15-BMI relationship. For the sake of clarity, we found that FAB15 exerted an indirect negative effect on BMI, which was partially explained by the negative association between FAB15 and BUT-AV. In other words, more performing executive functioning predicted a decrease in BMI that was partially due to the mitigation of avoidance behaviors.

## Discussion

With the aim to disentangle the unclear relationship between EFs and obesity (18–20, 30, 96, 97), the present study was designed to investigate the possible associations between EFs, body image and BMI in a nonclinical sample. Results from a preliminary multiple linear regression analysis showed that BMI was predicted by anthropometric variables (i.e., age and years of education), FAB15 score, in addition to both BUT-BIC and BUT-AV scores. *Via* a subsequent mediation analysis, we found that the negative association between EFs and BMI was partially explained by reduction of avoidance strategies.

Typically, avoidance is encountered in individuals with body image disorders. It leads people to avoid looking at themselves in the mirror and/or being looked at; in addition, thoughts concerned with alleged body defects significantly affect intimate, social, and work life. Avoidance was also associated with food choices (e.g., avoidance of sugary foods) and with interoceptive and emotional feelings inducing food ingestion (e.g., feeling of empty stomach or guilt after eating) (91).

To understand how EFs may interact with body weight and body image perception, examining the interaction between body weight and brain physiology is needed. Neuroimaging studies have shown hypoperfusion in the frontal territories and in the adjacent portions of the temporal and parietal cortices at increasing BMI levels (36–38). In addition, amygdala, ventral striatum, insula, and prefrontal regions (in particular, the orbitofrontal cortex and dorsomedial prefrontal cortex) functionally interact in order to promote approach conditions/behaviors, avoidance strategies/behaviors and decision-making processes (98, 99).

Decision making is a dynamic process, achieved after comparing and agreeing the current internal state with the expected one. Individuals constantly arbitrate between potential negative and/or rewarding outcomes when faced with a conflicting context. In this regard, excessive avoidance is presumably associated to overactivity of the amygdala and/or insular regions (100, 101). The increased signals related to the salience of anticipated emotional and interoceptive stimuli might decrease orbitofrontal activity that is, instead, aimed at integrating these signals. In this context, other relevant contingent information might be underestimated. Interestingly, a dysfunctional representation of approach conditions may be likely related to abnormal neural activity in the ventral striatum (102, 103). Attenuation of striatal activity may result in decreased motivation during effort-based decision-making tasks. Conversely, increased striatal activation could be related to overrepresentation of approach appraisal (104, 105).

The concomitant presence of conflicting motivational stimuli, such as approach- and avoidance-related stimuli, might overload the orbitofrontal cortex, leading to increased reaction time, as revealed during some decision-making paradigms, particularly those involving both reward and punishment, such as risk-based paradigms (106–108).

According to the “corticolimbic disconnection hypothesis” (109, 110), fronto-limbic suppressive mechanisms generate a state of emotional numbness. In particular, the prefrontal cortex would interact with the anterior cingulate cortex and amygdala, determining low emotionality, attentive difficulties, autonomic mitigation, and indifference to pain (100, 101, 111, 112). In addition, hypoactivity of posterior parietal regions has been associated with deficits in processing and integrating somatosensory information (111, 113–115) and low self-awareness (116).

The somatosensory pathways are involved in both conscious perception/recognition of one’s own body (i.e., the body image) (117) and in the body schema construction (118), i.e., a dynamic representation of one’s own body used to drive actions (119, 120). The terms body image and body schema are used to refer to two different dimensions of body representation, aware and unaware, respectively (121). Body image refers to “the body we perceive” and the conscious appraisal of one’s physical appearance (perceptual, cognitive, emotional) that is differentiated from the environment; conversely, body schema refers to “the body we act with” connected to the unconscious sensorimotor and postural control of one’s own body (sensory-motor capacities that function in communion with environment). However, the distinction is still unclear, and these terms have been and continue to be used in an arbitrary way (121).

In addition, the posterior parietal cortex, in concert with frontal cortex, the posterior insula and the angular gyrus, plays a pivotal role in integrating different input signals related to self-awareness in terms of enteroception, feelings of agency, and visceral sensations (122–129).

Overweight and obese subjects are more likely to misperceive their body features, i.e., they tend to underestimate (130, 131) or overestimate (60, 61, 127) their whole body or selective body parts (55, 62, 132, 133), particularly when these are perceived as unattractive (134–136).

Recently, the mirror exposure therapy (MET) has been proposed as an effective treatment for body image dissatisfaction (137, 138). MET may normalize interpretive biases by training individuals to assess their bodies in an objective, affectively neutral, or positive, manner (139, 140). Furthermore, MET may act by redirecting the focus of attention away from the negative body parts to the more balanced (141, 142), thereby gradually reducing self-focused attention. In other words, MET behaves as an exposure therapy by enhancing extinction through formation of a new safety memory that attenuates the negative response and/or through habituation (143, 144).

Our results showed that EFs exert a negative effect on BMI, which is partly justified by a detrimental action on avoidance, a subdimension of the body image construct. Although the relationship between body dissatisfaction and EFs is poorly investigated, our results suggest the involvement of executive abilities in modulating the cognitive processes underlying eating behavior and body weight control. In particular, EFs seem to indirectly influence the motivational systems involved in the processes of approaching/avoiding body image. Executive domains embrace the processes by which goal-directed actions are carried out, such as maintaining salient information in working memory and inhibiting non-goal-related responses (21–24). Successful self-regulation implies that individuals not only have sufficient motivation to reduce the discrepancy between the actual body image and the standard they are pursuing, but also the ability to achieve this reduction in the discrepancy. The ability to self-regulate is strongly related to EFs.

In a scientific context in which the link between EFs and obesity needs to be further explored, our findings may contribute to extend the debate on the matter.

## Data availability statement

The raw data supporting the conclusions of this article will be made available by the authors, without undue reservation.

## Ethics statement

The studies involving human participants were reviewed and approved by University of Campania “Luigi Vanvitelli”. The patients/participants provided their written informed consent to participate in this study.

## Author contributions

Conceptualization, MLM, AM, CRI and IV. methodology, MLM and CRI. software, CRI. validation, AM and IV. formal analysis, MLM and CRI. investigation, MLM, CRI, RP, MRS, MF, AC, and FS. resources, AM, GDM, IV and VM. data curation, CRI, RP, MRS, FS and GDM. writing—original draft preparation, MLM, AM, CRI and IV. writing—review and editing, MLM, CRI, IV and AM. visualization, MRS, MF, AC and FS. supervision, MLM, CRI, IV, AM, GMe and VM. project administration, MLM, GMe, IV and VM. All authors contributed to the article and approved the submitted version.

## Conflict of interest

The authors declare that the research was conducted in the absence of any commercial or financial relationships that could be construed as a potential conflict of interest.

## Publisher’s note

All claims expressed in this article are solely those of the authors and do not necessarily represent those of their affiliated organizations, or those of the publisher, the editors and the reviewers. Any product that may be evaluated in this article, or claim that may be made by its manufacturer, is not guaranteed or endorsed by the publisher.
